# Integrating Radio-Over-Fiber Communication System and BOTDR Sensor System

**DOI:** 10.3390/s20082232

**Published:** 2020-04-15

**Authors:** Wai Pang Ng, Nageswara Lalam, Xuewu Dai, Qiang Wu, Yong Qing Fu, Peter Harrington, Nathan J. Gomes, Chao Lu

**Affiliations:** 1 Department of Mathematics, Physics and Electrical Engineering, Northumbria University, Newcastle upon Tyne NE1 8ST, UK; xuewu.dai@northumbria.ac.uk (X.D.); qiang.wu@northumbria.ac.uk (Q.W.); richard.fu@northumbria.ac.uk (Y.Q.F.); peter.harrington@northumbria.ac.uk (P.H.); 2 National Energy Technology Laboratory (NETL), Pittsburgh, PA 15236, USA; Nageswara.Lalam@netl.doe.gov; 3 School of Engineering and Digital Arts, University of Kent, Canterbury CT2 7NZ, UK; N.J.Gomes@kent.ac.uk; 4 Department of Electronic and Information Engineering, Hong Kong Polytechnic University, Hong Kong; enluchao@polyu.edu.hk

**Keywords:** radio-over-fiber (RoF), distributed fiber sensor, BOTDR

## Abstract

In this paper, we propose and experimentally demonstrate for the first time, the integration of a radio-over-fiber (RoF) communication system and a Brillouin optical time-domain reflectometry (BOTDR) distributed sensor system using a single optical fiber link. In this proof-of-concept integrated system, the communication system is composed of three modulation formats of quadrature phase-shift keying (QPSK), 16-quadrature amplitude modulation (16-QAM) and 64-QAM, which are modulated onto an orthogonal frequency division multiplexing (OFDM) signal. Whereas, the BOTDR sensor system is used for strain and/or temperature monitoring over the fiber distance with a spatial resolution of 5 m using a 25 km single-mode silica fiber. The error vector magnitude (EVM) is analyzed in three modulation formats in the presence of various BOTDR input pump powers. Using QPSK modulation, optimized 18 dBm sensing and 10 dBm data power, the measured EVM values with and without bandpass filter are 3.5% and 14.5%, respectively. The proposed system demonstrates a low temperature measurement error (±0.49 °C at the end of 25 km) and acceptable EVM values, which were within the 3GPP requirements. The proposed integrated system can be effectively applied for practical applications, which significantly reduces the fiber infrastructure cost by effective usage of a single optical fiber link.

## 1. Introduction

Recent years have seen mobile communication technology growing rapidly, partly due to the huge demand for high data rate services. Fifth-generation (5G) communication network systems are being developed to achieve high data rates. Fourth-generation (4G) technology, such as long term evolution (LTE) and LTE-Advanced, has introduced a network architecture that includes an enhanced NodeB (eNB) and home eNB (HeNB) for both indoor and outdoor wireless applications, respectively [1,2]. In both 5G and 4G communication systems, quadrature amplitude modulation (QAM) technique permits a communication system to meet the data rates to match the 4G and 5G requirements [3,4].

On the other hand, structural health monitoring is a key element of safety and management of various infrastructures. Conventional fiber sensors, for instance, fiber Bragg grating (FBG) and long period Grating (LPG) sensors measure only at a specific location of interest. Since around 10 years ago, the use of Brillouin based distributed fiber sensor systems for structural health monitoring applications has increased rapidly [5,6], due to their high measurement range up to tens of kilometers. The familiar techniques in time-domain based Brillouin fiber sensors are; Brillouin optical time-domain reflectometry (BOTDR) [7] and Brillouin optical time-domain analysis (BOTDA) [8]. The BOTDR system is implemented using spontaneous Brillouin scattering (SpBS) which requires access to one end of the fiber. The BOTDA system uses stimulated Brillouin scattering (SBS) with counter-propagating continuous probe wave and access to both ends of the fiber is essential. Providing a new fiber infrastructure for a distributed sensing system comes at a huge cost with much complexity, which discourages their widespread use. Therefore, sharing an existing data transmission fiber infrastructure for distributed sensing offers cost-effectiveness and efficiency savings. For example, the railway industry uses a fiber infrastructure installed along the track-side for data transmission. The integration of distributed sensing system with the existing optical cable can be used for real-time condition monitoring of land-slides, track ballast, snowdrifts, flooding and line-side fire detection [9]. The integration of distributed sensing and active data transmission using a single optical fiber is an unexplored area of research.

In this paper, we demonstrate a proof-of-concept of simultaneous integration of fiber communication system and the BOTDR sensor system using a single optical fiber, demonstrating the performance of both systems experimentally. For the fiber communication system, the error vector magnitude (EVM) is analyzed for three different modulation formats of quadrature phase-shift keying (QPSK), 16-QAM and 64-QAM at different data and sensing system powers using a 25 km single-mode silica fiber. In order to evaluate the BOTDR sensor system performance, the temperature effects have been measured and the temperature errors evaluated. To demonstrate the proof-of-concept, we used a single 25 km fiber spool in the experiments. However, the sensing range can be further improved by in-line erbium-doped fiber amplifier (EDFA) [10] and Raman amplification [11,12], while choosing the optimized larger wavelength spacing between the data and sensing systems. It is significant to remark on certain previous works, such as Cucka et al. [13], which validated a simulation performance based on VPI photonics for phase-sensitive optical time-domain reflectometry (OTDR) into dense wavelength division multiplexing (DWDM) fiber data transmission. In 2017, Munster et al. [14] demonstrated a phase-OTDR system into a dual-polarization QPSK based data system and investigated the Q-factor performance influenced by the phase-OTDR system power. However, in their work, the sensing performance was not investigated in the presence of active data transmission. Therefore, the implementation and purposes of the system proposed in this paper are significantly different. Essentially, the proposed system is based on BOTDR and QAM over OFDM based communication system (aligns to 4G, 5G and beyond implementation [15]), where its performance is experimentally characterized by the simultaneous temperature and communication system EVM measurements.

## 2. Integration of Fiber Communication System and BOTDR Sensor System

**Figure 1:**
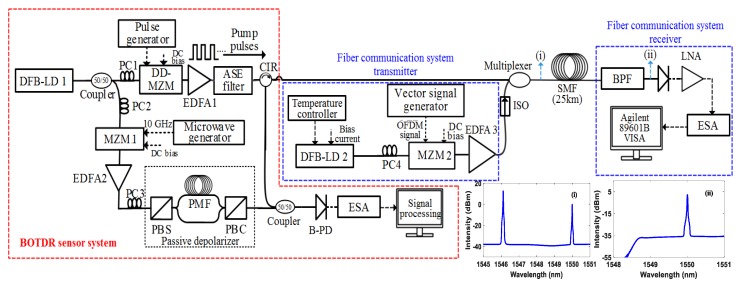
Experimental configuration for the integration of Brillouin optical time-domain reflectometry (BOTDR) sensor system and fiber communication system. (DFB-LD = distributed-feedback laser diode, PC = polarization controller, MZM = Mach-Zehnder modulator, DD-MZM = dual drive-MZM, EDFA = erbium-doped fiber amplifier, ASE = amplified spontaneous emission, PBS = polarization beam splitter, PMF = polarization maintaining fiber, PBC = polarization beam combiner, CIR = circulator, B-PD: balanced photodetector, ESA = electrical spectrum analyzer SMF = single-mode fiber, ISO = isolator, BPF = band pass filter, LNA = low noise amplifier).

The experimental configuration for the integrating BOTDR sensor system and fiber communication system is shown in Figure 1. The BOTDR sensor system is illustrated in a red dashed box of Figure 1. A distributed feedback (DFB) laser at a 1546.12 nm wavelength and 100 kHz linewidth is used. Thereafter using a 50/50 coupler, the signal is split into two pathways where the upper path signal is used for the optical pulses and the lower path is uses for the local oscillator (LO). The electrical pulses (generated from the pulse generator) are modulated into optical pulses using a dual-drive Mach-Zehnder modulator (DD-MZM). Using the Erbium-doped fiber amplifier (EDFA), the signal is amplified to an optimized power level. The amplified spontaneous emission (ASE) filter (pass-band: 1550 ± 5 nm) is employed to exclude the ASE noise generated from the EDFA. Using MZM 1, the LO signal is modulated by a 10 GHz frequency, which is upshifted and downshifted by 10 GHz from the pump signal. The polarization noise is the dominant noise source in Brillouin based distributed fiber sensor systems, therefore we used a simple and low-cost passive depolarizer to reduce the polarization noise [16]. The backscattered signal from the coupler is connected to the balanced photodetector (PDB470C, 400 MHz bandwidth) and analyzed using an electrical spectrum analyzer (ESA) in zero span mode.

The experimental setup of the fiber communication system transmitter and receiver is illustrated in Figure 1 (showed in blue dashed box). A DFB laser diode provides a continuous wave (CW) at 1550 nm wavelength with a temperature controller, to maintain a constant laser temperature at 25 °C. The laser emission is then externally modulated using an MZM (Photline, MXAN-LN-20). The polarization controller (PC) is used before the MZM to attain maximum power at the MZM output. In this configuration, the data signal is generated by a vector signal generator (Agilent-E4438C) with three modulation formats of QPSK, 16-QAM and 64-QAM with OFDM signal independently. The vector signal generator produces a real-time OFDM signal with a carrier frequency of 2.6 GHz and the channel bandwidth of 10 MHz via Agilent Signal Studio^TM^ software [17]. After the MZM, the signal is then amplified to the required launch power using an EDFA and then transmitted over a 25 km single-mode silica fiber link. At the receiver end, the RF signal is detected by a PD (Thorlabs, PDA8GS) and amplified using a low noise RF amplifier (LNA, PE15A1008). Thereafter, the detected signal is analyzed using an electrical spectrum analyzer (Agilent-9020A-MXA), which is automated using Agilent 89601B VSA software. Using the VSA software, the EVM of detected symbols is estimated [18]. The system parameters used in the experiments are defined in Table 1.

Since the BOTDR system requires only single end access of the fiber, the complete BOTDR sensor system is located at one end, as the input pump signal and backscattered signal operate at the same end of the fiber. The pulse width was set to 50 ns, corresponding to 5 m spatial resolution and the pulse period was 255 µs. The communication system transmitter was composed of a QPSK, 16-QAM and 64-QAM independently with bit rates of 16, 33 and 50 Mbps, respectively. It is significant to employ an isolator after the communication system transmitter; therefore, the signal reflections do not influence the data transmitter operation. The data signal and the sensor signal are simultaneously sent through a multiplexer and then to a 25 km single-mode silica fiber. At the receiver, a band-pass filter (BPF) is used to eliminate the sensor signal and extract the communication data signal. In the experiment, we employ a 1550 ± 2 nm polarization maintaining BPF (AFR’s-1550 nm bandpass filter) with a low insertion loss of 1 dB and maximum optical power handling of 300 mW. Therefore, data detection at the receiver will not be influenced by the sensor system signal. The measured optical spectrums after the multiplexer and BPF are illustrated in Figure 1 inset.

## 3. Results and Discussions

**Figure 2:**
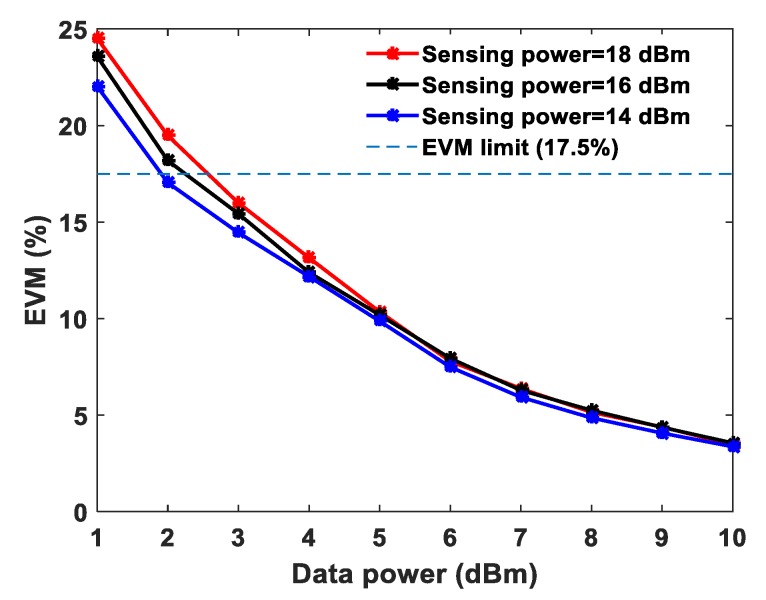
Measured error vector magnitude (EVM) of quadrature phase-shift keying (QPSK) for different data system powers at a fixed sensing power.

In order to evaluate the data system performance in the presence of sensing system power, the EVM is analyzed at fixed sensing powers of 14, 16 and 18 dBm. In our experiments, the EVM values are estimated using Agilent VSA software, which is connected to the electrical spectrum analyzer. Firstly, the EVM performance is analyzed based on QPSK modulation using a bandwidth of 10 MHz at 2.6 GHz carrier frequency. Figure 2 shows the measured EVM values against the different data system powers.

**Figure 3:**
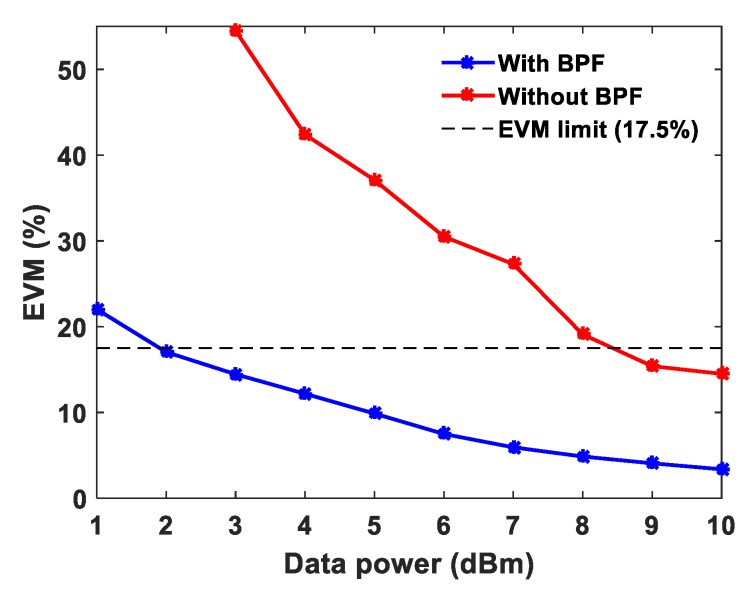
Measured EVM of QPSK modulation with and without band-pass filter (BPF) for various data system powers and fixed sensing power of 18 dBm.

**Figure 4:**
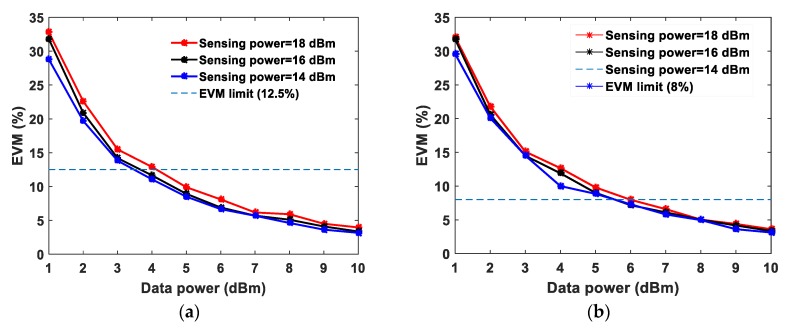
Measured EVM for various data system powers at a fixed sensing power, (**a**) 16-QAM (**b**) 64-QAM with 10 MHz bandwidth.

**Figure 5:**
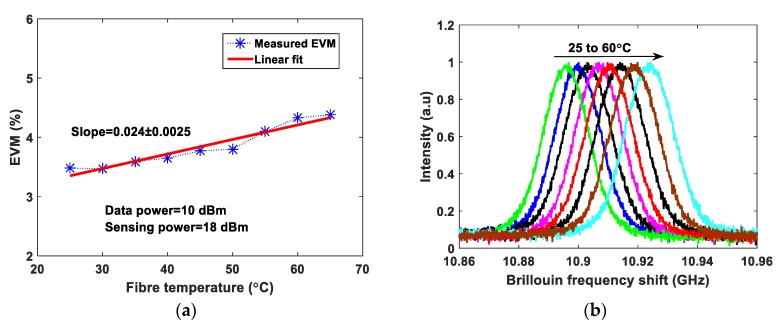
The temperature effects on (**a**) data system EVM of 64-QAM (**b**) sensing system Brillouin gain spectrum (BGS) response.

The EVM values at 1 dBm data power are ~24.5%, ~23.5% and ~22.1% obtained with the sensing powers of 18 dBm, 16 dBm and 14 dBm, respectively. The optimum sensing power is 18 dBm for a 25 km sensing fiber length [13]. At 10 dBm data system power, the measured EVM values are approximately the same at ~3.5% for the three different sensing system powers. The interference of the sensor signal and communication signal degrades the communication system performance, therefore, employing a BPF before the photodetector at the communication system receiver end is essential. Unfortunately, due to limited narrow band-pass filter availability, the wavelength spacing has been chosen in the experiments accordingly. The wavelength spacing between the distributed sensor and communication system can be closer to investigate the cross-impact on both the systems. This paper presents a proof-of-concept and feasibility of such emerging systems integration sharing a single fiber cable. In order to demonstrate the need for the optical BPF and mitigate the cross-interference in the proposed system, the EVM is analyzed with and without BPF at a fixed sensing system power of 18 dBm with QPSK modulation and the measured EVM values are shown in Figure 3. At 5 dBm data power the measured EVM values with and without BPF are 9.87% and 37.1%, respectively. Whereas, at 10 dBm data power the measured EVM values with and without BPF are 3.5% and 14.5%, respectively as shown in Figure 3. Without BPF, the sensing system power significantly influences the data signal, degrading the EVM performance. Thereafter, the EVM values are measured and analyzed for 16-QAM and 64-QAM modulation formats independently at fixed sensing powers, as illustrated in Figure 4a,b, respectively. In both modulation formats, at a 10 dBm data system power, the EVM values are approximately the same for various sensing powers. The obtained EVM values for 16-QAM and 64-QAM at 10 dBm data system power are ~3.4% and ~3.6%, respectively. The temperature effects have been measured in both data system and sensing system. The whole 25 km fiber was kept in a temperature-controlled oven and various temperatures were applied. The data power and sensing system powers are fixed at 10 dBm and 18 dBm, respectively. The EVM values of the data system (64-QAM) and Brillouin gain spectrum of the sensing system are measured simultaneously and the results are illustrated in Figure 5a,b. The measured EVM values are fitted with a linear curve and the obtained slope is 0.024 ± 0.0025%/°C. At room temperature (~25 °C), the obtained BFS is 10.89 GHz and the slope (temperature sensitivity) is 1.07 ± 0.013 MHz/°C.

**Figure 6:**
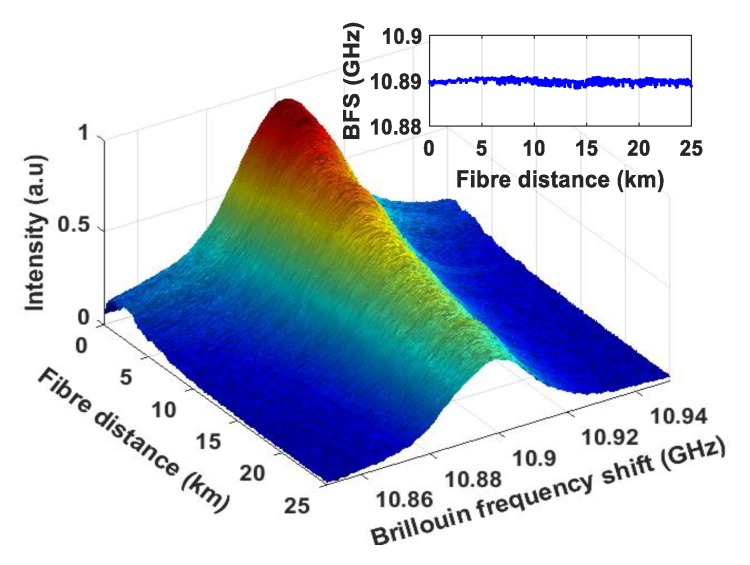
Three-dimensional Brillouin gain spectrum along the 25 km fiber distance. Inset: BFS distribution along the fiber distance.

At room temperature (~25 °C) and at a fixed data system power (10 dBm) and a sensing power (18 dBm), the three-dimensional Brillouin spectrum is obtained and shown in Figure 6. Sweeping the Brillouin frequencies from 10.85 GHz to 10.95 GHz and a frequency step of 1 MHz, the three-dimensional spectrum was mapped. Using the Lorentz curve fitting, the peak Brillouin frequency over the 25 km fiber is illustrated in Figure 6 inset.

**Figure 7:**
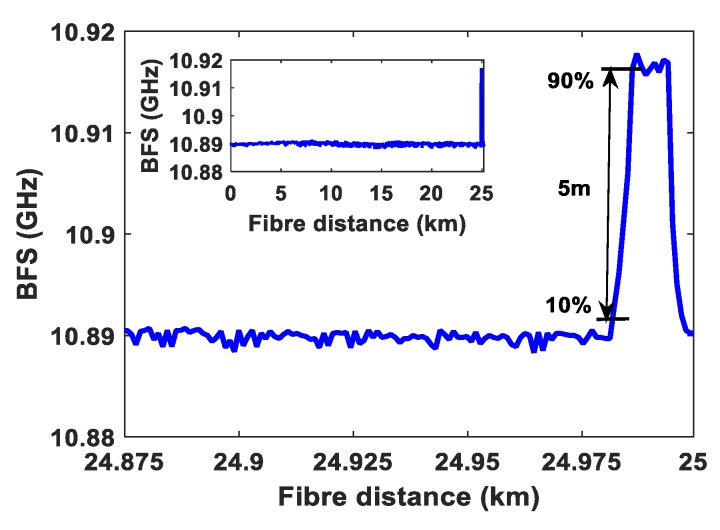
BFS distribution of 50 °C temperature on 20 m fiber, (inset: BFS over the whole length of the fibe).

Finally, in order to evaluate the sensing system performance and spatial resolution in the presence of active data transmission, a 20 m fiber length at end of the fiber of the 25 km fiber is placed in the temperature-controlled oven and the rest of the fiber remains at the room temperature of 25 °C. The temperature was set at 50 °C in the oven. The peak Brillouin frequency over the 25 km optical fiber is illustrated in Figure 7 inset. The spatial resolution of 5 m is attained as shown in Figure 7, which confirms the 50 ns pulse width used in the BOTDR sensor system. For a 25 °C temperature change on 20 m long fiber at the fiber end, the estimated BFS change is 26.75 MHz, as the sensing fiber sensitivity is 1.07 MHz/°C. From Figure 7, the measured BFS change is 27.28 MHz for a temperature change of 25 °C. Therefore, the BFS error is 0.53 MHz, which corresponds to a temperature error of ±0.49 °C. The results validate a precise temperature measurement without any effects originated from the data communication system.

## 4. Conclusions

We have proposed and experimentally demonstrated a proof-of-concept of integrating fiber communication system and BOTDR sensor system using a single 25 km optical fiber at two different dedicated wavelengths. The communication system EVM performance was investigated using different modulation formats of QPSK, 16-QAM, 64-QAM in the presence of various BOTDR sensor system optimized power levels. The EVM performance with and without BPF before the photodetector of the data system receiver is demonstrated. Using a QPSK modulation and 18 dBm sensing power and at 10 dBm data power the measured EVM values with and without BPF are 3.5% and 14.5%, respectively. While the BOTDR temperature sensing performance has been investigated in the presence of data system signal. As a result, the measured temperature error is ±0.49 °C when the applied temperature is 50 °C at the end of the sensing fiber., Therefore, the proposed system demonstrates a low temperature measurement error ( ±0.49 °C) and acceptable EVM values, which were within the 3GPP requirements, hence demonstrating the feasibility for practical applications. For further study, the proposed system configuration can be investigated for narrow wavelength spacing between data and sensor system laser wavelengths. The proposed system can be further exploited into DWDM and CWDM based communication system to improve the system performance in terms of larger bandwidth/higher data rate [15]. Furthermore, the BOTDR sensor system can be integrated with other emerging techniques, such as Raman amplification, pump pulse coding and wavelength diversity techniques [19] for further improvement in sensor system performance.

## Figures and Tables

**Table 1:** Experimental system parameters.

Fiber Communication System		BOTDR Sensor System	
Parameter	Value	Parameter	Value
optical wavelength	1550 nm	optical wavelength	1546.12 nm
optical power	0 to 10 dBm	input pump power	14, 16 and 18 dBm
modulation scheme	QPSK, 16-QAM and 64-QAM	DD-MZM bandwidth	12 GHz
bit rate (at channel bandwidth: 10 MHz)	QPSK = 16 Mbps 16-QAM= 33 Mbps 64-QAM = 50 Mbps	pulse width, period	50 ns, 255µs
baseband multiplexing	OFDM	pulse repetition rate	4.2 kHz
carrier frequency	2.6 GHz	pulse amplitude	4 Vpp
channel bandwidth	10 MHz	EDFA 1 gain, noise figure	30 dB, 6 dB
RF power	0 dBm	EDFA 2 gain, noise figure	28 dB, 5.5 dB
MZM bandwidth	20 GHz	ASE filter passband	1545–1555 nm
EDFA gain, noise Figure	28 dB, 4.8 dB	PMF length	5 km
